# Mathematical modeling and mechanisms of HIV latency for personalized anti latency therapies

**DOI:** 10.1038/s41540-025-00538-6

**Published:** 2025-06-12

**Authors:** Gianmarco Rasi, Elena Emili, Jessica M. Conway, Nicola Cotugno, Paolo Palma

**Affiliations:** 1 Research Unit of Clinical Immunology and Vaccinology, Bambino Gesù Children’s Hospital, IRCCS, 00165 Rome, Italy; 2 PhD Program in Immunology, Molecular Medicine and Applied Biotechnology, University of Rome “Tor Vergata”, 00133 Rome, Italy; 3 Department of Systems Medicine, University of Rome “Tor Vergata”, 00133 Roma, Italy; 4 Department of Mathematics and Center for Infectious Disease Dynamics, Pennsylvania State University, University Park, PA USA

**Keywords:** Biochemical networks, Stochastic modelling

## Abstract

Combination antiretroviral therapy controls human immunodeficiency virus-1 (HIV) but cannot eradicate latent proviruses in immune cells, which reactivate upon treatment interruption. Anti-latency therapies like “shock-and-kill” are being developed but are yet to succeed due to the complexity of latency mechanisms. This review discusses recent advances in understanding HIV latency via mathematical modeling, covering key regulatory factors and models to predict latency reversal, highlighting gaps to guide future therapeutic approaches.

## Introduction

**Fig. 1:**
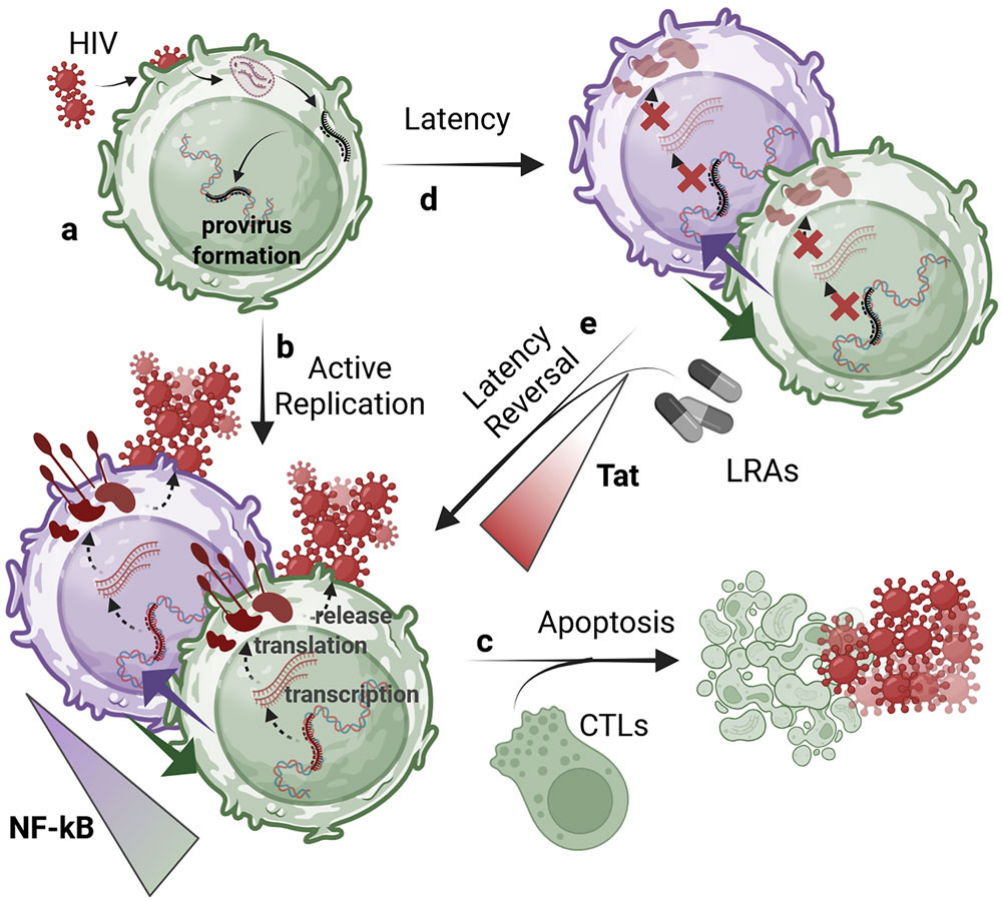
HIV developmental fate upon CD4 + T cell infection. **a** HIV cell infection, proliferation, and latency reversal. Once HIV enters a susceptible cell (i.e., active CD4 + T-lymphocyte), is reverse-transcribed, and integrates within the host genome, forming a provirus. **b** Transitioning into active-replication. Upon sufficiently-high transactivator of transcription (Tat) production, a provirus enters active replication, forcing the host-cell to fabricate and release virions. **c** host-cell loss and HIV proliferation. The accumulation of viral products leads to host-cell apoptosis within ~40 *h* of active replication. **d** Transitioning into latency. Conversely, upon low Tat concentrations, the provirus remains silent. As opposed to what was initially believed, active replication and latency can be detected both in active and resting lymphocytes, represented by green and purple cells, respectively. **e** Latency-reversal. Upon increased Tat levels a dormant provirus activates from latency. The shock-and-kill therapy employes latency reversing agents to enhance Tat production and facilitate latency-reversal. [Created in BioRender. Palma, P. (2025) https://BioRender.com/0fw3cwk].

HIV latency remains a major barrier to achieving a HIV cure. When HIV enters a susceptible cell, its RNA is reverse-transcribed and integrates semi-randomly^1^ into the host genome, forming a provirus [Fig. 1a]. The provirus can follow one of two developmental fates: active-replication or latency. Actively replicating proviruses sustain a relatively high gene-expression activity, driving HIV proliferation and acquired immunodeficiency syndrome (AIDS) progression. In particular, after ~40h^2^ of activity, the accumulation of viral products within the intracellular space drives host-cell lysis, allowing the hundreds of assembled virions^3^ to diffuse and infect nearby susceptible cells [Fig. 1b, c]. Antiretroviral therapy (ART) acts disrupting the critical steps of the HIV replication program, interrupting viral proliferation, and allowing the progressive depletion of productively-infected cells. However, even after decades of fully-effective treatment, ART interruption causes almost certain viremia rebound within 2-8 weeks^4,5^. Evidence suggest that this rebound is due to long-lived proviruses activating from latency^6–8^ [Fig. 1d]. Latent proviruses are unbale to sustain significant viral activity^9^ due to molecular blocks hindering their gene-expression program. However, their dormancy is reversible and they switch to active replication as soon as the blocks are lifted. Moreover, as long as the provirus is silent, the hosting cell is virtually indistinguishable from its healthy counterpart, allowing the provirus to survive without replicating. Therefore, given their ability to potentially ignite HIV proliferation after years of dormancy, latent proviruses represent the main obstacle for curing HIV^10,11^.

Curative strategies now aim at reducing the *reservoir* of latent HIV proviruses. The most studied method is called *shock*-*and*-*kill*^12^. This method implies using molecules known as latency reversing agents (LRAs) to disrupt the mechanisms that keep the provirus silent (*shock* phase). Then, once activated, the provirus would be cleared by cytolytic effects and immune response (*kill* phase) [Fig. 1e, f]. Developing effective LRAs is critical. It requires identifying the molecular factors regulating proviral latency and their mechanisms of action [Tables 1–3].

**Table 1:** Models addressing the basal (host-driven) HIV gene-expression activity

	Study	Aims	Data	Results
**Basal HIV Gene-Expression**	A. Singh et al.^27^	Understand the mechanisms underlying basal HIV gene-expression dynamics.	Flow cytometry analysis of GFP expression, sampled from 30 Jurkat T-cell iso-clones infected with a single LTR-GFP HIV model vector with diverse IS.	(**i**) Provirus-specific basal HIV gene-expression is highly variable (noisy); (**ii**) The observed variability is poorly explained by the constitutive LTR model. Instead, it is well reproduced by a two-state (on/off) random telegraph model underlying random bursting activity; (**iii**) During host phase, the LTR produces bursts with average size of 2–10 mRNA.
	A. Singh et al.^75^	Understand which viral gene-expression model (constitutive vs bursting) prevails during the host-phase	Flow cytometry analysis of GFP expression, sampled from Jurkat T-cells iso-clones infected with a single LTR-GFP and LTR-mCh HIV model vector with diverse IS.	(**i**) promoter toggling between an active and inactive state is the main source of noise in basal HIV gene-expression.
	R. D. Dar et al.^45^	Understand what viral gene-expression model (i.e., constitutive, bursting) better explains provirus-specific basal gene-expression activity.	Flow cytometry analysis of GFP expression, sampled from 8000 Jurkat T-cells iso-clones infected with a single LTR-GFP HIV model integrated in a different genomic *locus*.	(**i**) Promoter toggling between an active and inactive state is the main source of noise in basal HIV gene-expression; (**ii**) The MME at the provirus IS modulates both burst frequency and size; (**iii**) Below an average gene-expression level, MMEs modulates only bursting frequency, whereas above such threshold, MMEs regulate only burst size; (**iv**) Transcriptional activators (i.e., TSA, TNF-*α*) regulate burst frequency and size along a provirus-specific constrained region.
	K. Tantale et al.^28^	Understand the mechanisms underlying basal HIV transcription, and the host factors modulating those mechanisms.	Single-molecule RNA fluorescence in situ hybridization (smFISH) analysis of isogenic MCP-GFP-expressing HeLa Flp-in H9 cells infected with an MS2-labeled HIV model vector in the same IS, high Tat production.	(**i**) HIV mRNA is produced by closely-spaced RNAPII *convoys* allowed by a Mediator-driven mechanism; (**ii**) RNAPII convoys are spaced by ~hundred nucleotides due to DNA torsional stress; (**iii**) The LTR displays stochastic bursting activity on two time scales, referred to as multi-scale bursting: The first underlie the rate of RNAPII convoys, the second the LTR activation rate.
	K. Tantale et al.^29^	Understand how RNAPII pausing regulate basal transcription.	smFISH analysis of three distinct isogenic MCP-GFP-expressing HeLa Flp-in H9 cell lines infected with an 128xMS2-labeled HIV model vector, characterized by low, medium, and high Tat expression.	(I) RNAPII enter a long-lived pause (>20 min) in silent LTR, limiting viral transcription; (ii) RNAPII pausing is not obligatory but stochastic. Only a small fraction of RNAPII undergo long-lived pausing in basal regime;

**Table 2:** Models addressing the viral protein Tat-circuit architecture

	Study	Aims	Data	Results
**Tat feedback-loop & Proviral Fate**	L. Weinberger et al.^20^	Understand the factors and mechanisms regulating HIV developmental fate.	GFP expression sampled from Jurkat T-cells, infected with a single LTR-GFP-IRES-Tat (LGIT) HIV model vector.	(**i**) Proviral developmental fate (active replication vs latency) is regulated by Tat. (**ii**) Proviruses showing a relatively high basal gene-expression rate (high Tat), experience active replication; (i**ii**) Provirus clones showing relatively low basal gene-expression rate (low Tat) face a stochastic decision between high and negligible activity (PheB);
	B. S. Razooky et al.^18^	Understand the relationship between proviral fate (active vs latent) and host-cell state (active vs resting)	GFP expression sampled from Jurkat and CEM T-cells infected with HIV-d2GFP virus env-mutated (avoid expansion).	(**i**) The LTR is intrinsically capable of generating bimodal ON/OFF expression, even in the absence of Tat; (**ii**) Tat slows LTR toggling, shifting, and expanding the regime of LTR bimodality ultimately characterizing a stabler active replication regime;
	L. Weinberger et al.^25^	Understand the mechanisms by which Tat-positive feedback is counteracted, allowing the existence of a stable transcriptionally-off state (latency).	GFP expression sampled from Jurkat cells, infected with LTR-GFP and LTR-GFP-IRES-Tat (LGIT) HIV model vector.	(**i**) The Tat-feedback circuit lacks bi-stability and self-cooperativity; however, it exhibits pulsatile activity patterns triggered by stochastic basal activity; (**ii**) An enzymatic Tat-resistor reduces the Tat-amplification susceptibility to transcriptional noise, explaining the HIV off (latent) state and the pulsatile HIV gene-expression activity.
	K. H. Aull et al.^58^	Understand the mechanisms by which the Tat-positive feedback is counteracted, allowing the existence of a stable transcriptionally-off state (latency).	flow cytometry and single-cell imaging	(**i**) The Tat-feedback circuit exhibits a transient threshold lasting ~40 h before disappearing (**ii**) whose lifetime is shortened by promoter activation;
	L. Weinberger et al.^26^	Understand how Tat-feedback strength modulates the pulsatile HIV gene-expression dynamics.	GFP expression sampled from TNF-*α*-stimulated^76,77^ (10 ng/ml) Jurkat T-cells (J-Lat full-length clone 10.6 ^74^) infected with a single LTR-GFP or LTR-GFP-IRES-Tat (LGIT) HIV model vector.	(**i**) Tat positive-feedback extends viral gene-expression lifetime 2-6 fold. (**ii**) weak Tat-amplifications provide a shortened gene-expression pulse, favoring the latent phenotype.
	B. S. Razooky et. al. ^17^	Understand the relationship between proviral fate (active vs latent) and host-cell state (active vs resting)	GFP expression sampled from activated (CD25^+^ CD69^+^) and resting (CD25^−^ CD69^-^) Jurkat and CEM T-cells infected with HIV-d2GFP virus env-mutated (avoid expansion).	(**i**) HIV gene-expression persists in acutely-infected cells after transitioning to their resting state (**ii**) Tat circuit is autonomous and regulates proviral fate (**iii**) Host factors stochastically ignite Tat amplification.

**Table 3:** Models addressing the effects of MME reconfiguration of HIV gene-expression

	Study	Aims	Data	Results
**Latency Reversal**	A. K. Chavali et al.^30^	Understand the factors and mechanisms related to the MME that modulate HIV gene-expression noise, eventually inducing latency-reversal upon induced MME reconfiguration.	LTR-driven GFP expression of 10^5^ HIV-infected Jurkat T-cell clones upon Aza and TNF stimulation.	(**i**) The two- and three-state LTR models captures the mechanisms by which basal HIV gene-expression may induce latency-reversal; (**ii**) The Fano-factor proves to be a useful noise metric to compare models’ prediction. (**iii**) The three-state LTR model well captures changes in basal viral activity after LRAs-driven MMEs reconfigurations.
	V. G. Wong et al.^31^	Understand the factors and mechanisms related to the MME that modulate HIV gene-expression noise, eventually inducing latency-reversal upon induced MME reconfiguration.	time-resolved, single-cell transcriptional data over multiple IS upon NF-κB stimulation.	(**i**) The TNF-induced increase of transcription variability at the provirus level is higher than its mean HIV transcripts count; (**ii**) TNF-induced NF-kb activation correlates with latency-reversal. (**iii**) NF-kb levels, must be combined with chromatin structure and RNAPII regulation to explain the observed provirus-specific variability
	Y. Cao et al.^34^	(**i**) Identify targetable key reactions critical for Tat-amplification to shorten stochastic delays and speed-up latency reversal; (**ii**) Build HIV gene-expression model for in-silico LRAs efficacy testing	Parameters estimates taken from the literature	(**i**) The Tat circuit exhibits a bimodal probability landscape, where a peak is associated with latency, and another with the active fate; (ii) Enhancing Tat acetylation may increase Tat and viral production; (**iii**) Increasing the binding affinity between the LTR and Tat may induce an easier transition to the viral-phase; (i) Adopting a modeling framework is a valid approach to search and discovery potentially effective therapeutic strategies and compounds

Initial observations suggested that proviral activation is a secondary effect of the host-cell immune-activation^13,14^. In other words, the state of a provirus depends on the state of its host^15^. However, efforts to reverse latency solely by altering the host-cell state has proven this principle to be inaccurate^16^. Proviruses do not always enter latency when the host-cell becomes quiescent, and some latent proviruses fail to activate when the immune system is active^16,17^. Mathematical modeling applied to single-cell experimental data has revealed that HIV self-regulates its transcription through an intrinsic mechanism based on the viral protein transactivator of transcription (Tat)^17–20^. These models demonstrated that HIV latency is not simply an “OFF” state but rather a dynamically maintained *basal regime*, characterized by low Tat levels. When Tat accumulates beyond a critical threshold, it triggers a positive feedback-loop that bypass host regulation^17,19,21^, amplifies viral transcription^18,20,22^, and shift the provirus into a robust *viral regime* [Fig. 1b, d]. Subsequent findings suggested that proviral fate is also regulated by post-transcriptional steps, including splicing, nuclear export, and translation^23^. Host factors such as RNA-binding proteins (e.g., MATR3, PTB, and PSF) and specific microRNAs act to sequester, degrade, or otherwise inhibit the efficient export and translation of HIV RNAs, thereby enforcing a block to virus reactivation^24^. According to these findings, the development of anti-latency strategies requires understanding of what host factors are regulating basal transcriptional and post-transcriptional activity, and how the Tat circuit reacts to basal activity^20,25–29^. Experimental assays that reveal the molecular microenvironment (MME) of integrated proviruses, together with HIV gene-expression models were developed to aid the design of personalized LRAs, predicting the efficacy of a given treatment through in-silico experiments^30–34^.

Recent literature reviews on HIV latency and anti-latency therapies primarily focus on molecular mechanisms, latency-reversing agents, and host factors driving viral persistence^35–38^. In contrast, our review adopts a systems biology perspective, examining HIV gene-expression as a circuit influenced by feedback loops, stochastic events, and regulatory motifs. In the first section of this review, we present the models developed to understand the basal HIV gene-expression dynamics^27–29^, and the main factors regulating its steps. In the second section we focus on the Tat circuit models, developed to elucidate how Tat reacts to the basal expression activity and identify the threshold to active-replication^17,18,20,25,26^. Building on the knowledge reviewed in these first two section we conclude showing the models developed to simulate perturbations to the HIV gene-expression circuit and predict the efficacy of anti-latency therapies, to proceed with their development^39–44^. By bridging virology with quantitative modeling, our interdisciplinary approach offers fresh insights into HIV latency mechanisms and provides a framework for optimizing therapeutic interventions, paving the way for future research at the nexus of systems biology and HIV treatment.

## Basal HIV gene-expression control

**Fig. 2:**
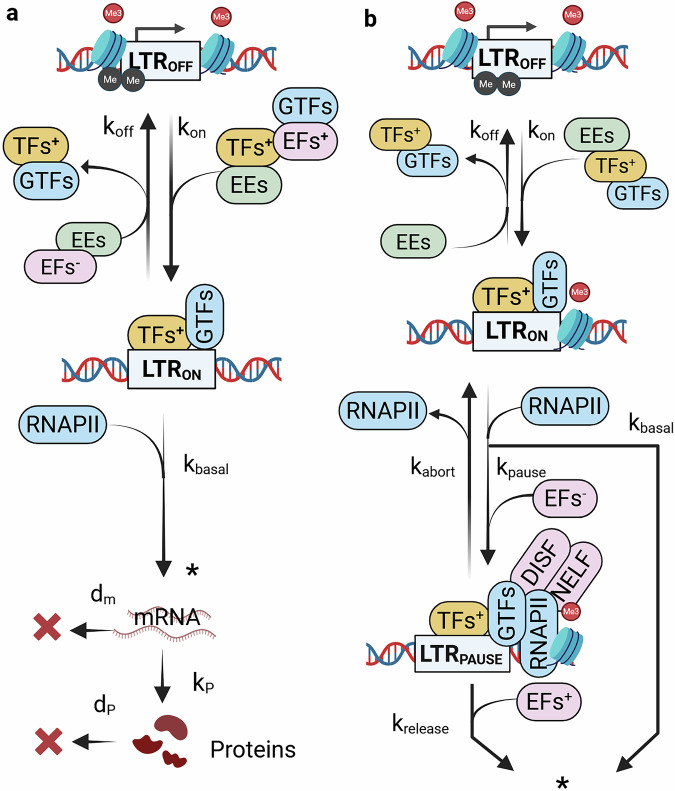
Basal HIV gene-expression models. **a** Bursting model. In this model the HIV promoter is allowed to switch between an active and inactive state, represented by *LTR*_*ON*_ and *LTR*_*OFF*_, respectively. The transition is driven by the rates *k*_*on*_ and *k*_*off*_, which represent the frequency of the molecular interactions dynamically shaping the LTR configuration that push the LTR towards a favorable or inhibited state, respectively. The LTR becomes active when the epigenetic editors (EE) allow for an open chromatin, and when the positive transcription factors (TFs), transcription machinery components (i.e., general transcription factors), and positive elongation factors (EF) are successfully recruited by the LTR. On the other hand, the LTR inactivates when the EE promote the formation of thick chromatin, when negative TF and/or EF are recruited and their positive counterparts are dismissed. When the LTR is transcriptionally-active, viral RNA is transcribed at rate *k*_*basal*_, representing successful recruitment of RNA polymerase II (RNAPII), and viral proteins are synthesized by it at rate *k*_*P*_. Simultaneously, viral transcripts and proteins are lost by degradation at rate *d*_*m*_, and *d*_*P*_, respectively. **b** Multi-bursting model. In this model the molecular interactions regulating the two transcriptional steps initiation and elongation are explicitly tracked. The LTR switches to its active state *LTR*_*ON*_ at rate *k*_*on*_, allowing transcription initiation, while it becomes transcriptionally inactive, *LTR*_*OFF*_ at rate *k*_*off*_. The LTR activates upon removal of epigenetic blocks, and recruitment of positive TF and GTFs. Upon RNAPII recruitment transcription may be paused at rate *k*_*pause*_, alternatively, it is successfully performed at rate *k*_*basal*_. The pausing takes place when elongation blocks are in place, such as recruitment of negative EF and/or nucleosome-1 positioning. In that case the LTR assumes a paused configuration *LTR*_*pause*_. When transcription is paused, RNAPII may abort transcription at rate *k*_*abort*_, or is released and produces elongated transcripts at rate *k*_*release*_. This dynamic results in the production of RNAPII convoys. The convoys were modeled considering the number of RNAPII involved (*N*_*pol*_), their time-spacing (*t*_*space*_), the elongation rate *v*_*el*_ and the time before completing an iteration, *t*_*proc*_, (convoys not depicted). [Created in BioRender. Palma, P. (2025) https://BioRender.com/6uc4cie].

Analyzes of basal HIV gene-expression reveal that viral activity is generally low on average and subject to significant intrinsic variability^27^ – often described as transcriptional noise. Knowing that latency exit occurs stochastically, understanding the mechanisms of transcriptional noise and its main regulatory factors could provide strategies to reverse latency. Early modeling efforts attributed this intrinsic noise to stochastic fluctuations inherent in the mRNA synthesis and degradation processes. This hypothesis led to the development of the *constitutive model* of HIV gene-expression^30^ [Fig. 2a]. In this model the HIV promoter—located in the 5’ long-terminal-repeat (LTR) – actively sustains HIV transcription (mRNA), leading to the synthesis of viral proteins (P).

1 $$\begin{array}{c}{\rm{LTR}}\mathop{\to }\limits^{{k}_{{\rm{basal}}}}{\rm{LTR}}+{\rm{mRNA}}\\ {\rm{mRNA}}\mathop{\to }\limits^{{k}_{{\rm{p}}}}{\rm{mRNA}}+{\rm{P}}\\ \begin{array}{c}{\rm{mRNA}}\mathop{\to }\limits^{{d}_{{\rm{m}}}}0\\ {\rm{P}}\mathop{\to }\limits^{{d}_{{\rm{p}}}}0\end{array}\end{array}$$

Although the constitutive model could not be validated biologically^27,45^, later studies proposed an alternative explanation. They suggested that the HIV promoter stochastically alternates between active and inactive states, promoting or inhibit transcription, respectively. This mechanism forms the basis of the two-state, or *random telegraph*, model of HIV expression^27,45–47^ [Fig. 2a]. In this model, stochastic transitions between LTR states generate randomly timed transcriptional bursts.

2 $$\begin{array}{c}{{\rm{LTR}}}_{{\rm{on}}}\mathop{\longrightarrow }\limits^{{k}_{{\rm{off}}}}{{\rm{LTR}}}_{{\rm{off}}}\\ {{\rm{LTR}}}_{{\rm{off}}}\mathop{\longrightarrow }\limits^{{k}_{{\rm{on}}}}{{\rm{LTR}}}_{{\rm{on}}}\\ {{\rm{LTR}}}_{{\rm{on}}}\mathop{\longrightarrow }\limits^{{k}_{{\rm{basal}}}}{{\rm{LTR}}}_{{\rm{on}}}+{\rm{mRNA}}\\ {\rm{mRNA}}\mathop{\longrightarrow }\limits^{{k}_{{\rm{p}}}}{\rm{mRNA}}+{\rm{P}}\\ {\rm{mRNA}}\mathop{\longrightarrow }\limits^{{d}_{{\rm{m}}}}0\\ {\rm{P}}\mathop{\longrightarrow }\limits^{{d}_{{\rm{p}}}}0\end{array}$$

These postulated LTR “ON” and “OFF” states could be explained biologically. In fact, fluctuating molecular interactions at the provirus integration site (IS) dynamically alter the LTR configuration, “locking” and “unlocking” transcriptional steps^28,29^. According to this view, identifying the factors interacting with the LTR and shaping its configuration would lead to regulatory factors of HIV transcription, and potential transcription-control strategies. These factors can be divided based on the transcriptional step they regulate (i.e., initiation, elongation). Transcription initiation requires an LTR surrounded by open chromatin, and successful recruitment of positive transcription factors (TFs) and general transcription factors (GTFs)^48^. This configuration allows RNA polymerase II (RNAPII) to dock and start processing the viral genome. Subsequently, transcription elongation requires RNAPII maturation and nucleosome-1 displacement^19,49,50^.

Subsequent studies using single-molecule RNA microscopy showed that the HIV promoter fluctuates over two independently-regulated inactive states, characterized by two distinct time scales^28^. The first time scale represents the time-delay between subsequent emergence of LTR configurations favorable for transcription initiation. Whereas the second time scale is shorter and represents the interval between elongation-free LTR configuration, characterizing the length of transcriptional bursts. It was also observed that transcriptional bursts are characterized by *convoys* of RNAPII that reinitiate transcription upon successful elongation. Moreover, the same studies demonstrated that RNAPII pausing is not obligatory but stochastic^29^. These observations led to the development of the multi-scale HIV bursting model^28,29^ [Fig. 2b].

3 $$\begin{array}{c}{{\rm{LTR}}}_{{\rm{on}}}\mathop{\to }\limits^{{k}_{{\rm{off}}}}{{\rm{LTR}}}_{{\rm{off}}}\\ {{\rm{LTR}}}_{{\rm{off}}}\mathop{\to }\limits^{{k}_{{\rm{on}}}}{{\rm{LTR}}}_{{\rm{on}}}\\ {{\rm{LTR}}}_{{\rm{on}}}\mathop{\to }\limits^{{k}_{{\rm{basal}}}}{{\rm{LTR}}}_{{\rm{on}}}+{\rm{mRNA}}\\ {{\rm{LTR}}}_{{\rm{on}}}\mathop{\to }\limits^{{k}_{{\rm{pause}}}}{{\rm{LTR}}}_{{\rm{pause}}}\\ {{\rm{LTR}}}_{{\rm{pause}}}\mathop{\to }\limits^{{k}_{{\rm{abort}}}}{{\rm{LTR}}}_{{\rm{on}}}\\ {{\rm{LTR}}}_{{\rm{pause}}}\mathop{\to }\limits^{{k}_{{\rm{release}}}}{{\rm{LTR}}}_{{\rm{on}}}+{\rm{mRNA}}\\ {\rm{mRNA}}\mathop{\to }\limits^{{k}_{{\rm{p}}}}{\rm{mRNA}}+{\rm{P}}\\ {\rm{mRNA}}\mathop{\to }\limits^{{d}_{{\rm{m}}}}0\\ {\rm{P}}\mathop{\to }\limits^{{d}_{{\rm{p}}}}0\end{array}$$

Despite the validity of the discussed HIV circuit models, recent studies showed that viral expression is also regulated during transcription completion, and post-transcriptional steps, including splicing and nuclear export^23^. These observations led to the development of more comprehensive models to understand how these steps affect the HIV expression dynamics and what are the involved molecular factors^51,52^.

**Fig. 3:**
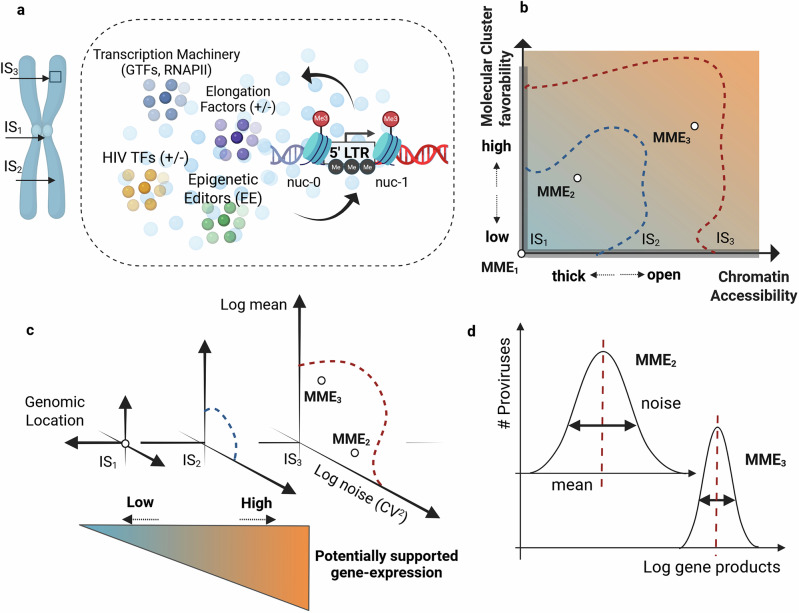
HIV molecular microenvironment and basal gene-expression potential. **a** Molecular microenvironment (MME) at the provirus integration site (IS). MMEs represent the epigenetic profile and the cluster of diffusing molecules at a given genomic location. The epigenetic profile is determined by the level of DNA methylation and nucleosomes positioning. The molecular cluster is composed of molecular groups, each regulating specific transcriptional and post-transcriptional steps. These main groups are the positive and negative transcription factors (TF^+^/ TF^−^) in yellow, positive, and negative elongation factors (EF^+^/ EF^−^) in purple, transcription machinery component in blue, and epigenetic editors (EE) in green. The cluster composition affects what reaction would likely rearrange the LTR configuration, and drive splicing and nuclear export. On the other hand, the epigenetic profile determines the chromatin thickness and affect the ability of surrounding molecules to interact with the LTR. **b** Heuristic representation of MME favorability and allowed MME configurations range. An MME characterized by open chromatin and a favorable molecular cluster composition would favor gene-expression activity. Different ISs would experience the formation of different range of MMEs, depending on the genomic entities populating the location. For example, IS_1_ represents a provirus integrated within a repressed region, characterized by the static unfavorable MME_1_. This provirus would eventually experience only a negligible expression activity. Conversely IS_2_ and IS_3_ are within expressed regions and their MME can assume different configurations, allowing, in turn, a variable range of gene-expression distributions. **c** Heuristic representation of basal HIV expression distribution vs IS genomic coordinates and basal HIV expression potential. The MME characterizing a provirus IS also determines the provirus’ basal activity. This activity is modulated both in terms of average and variability (noise). ISs within diverse genomic regions would be potentially exposed to different MMEs depending on the nature of the surrounding genomic entities. According to this view, the basal gene-expression potential – the area underlined by the possible MMEs configurations – is a single-values metric describing the viral expression energy associated with a single genomic location. **d** Holistic representation of basal HIV expression distributions vs MME. The MME at the provirus IS modulates the basal expression activity. [Created in BioRender. Palma, P. (2025) https://BioRender.com/4la57ft].

From a broader perspective, the LTR state and subsequent post-transcriptional steps, depend on the epigenetic profile and the composition of the molecular cluster fluctuating at the provirus IS. These factors are dynamically regulated by the host and characterize the provirus MME^45,53,54^ [Fig. 3a]. The host configures the MMEs along its genome to control the gene-expression activity of the genomic entities in-location. Therefore, MMEs at diverse genomic coordinates would be subjected to diverse regulatory inputs over time [Fig. 3b–d]. This explains why different IS shows different expression distributions and why non-specific stimuli, such as the host-cell immune activation, do not homogeneously affect all proviruses. Following this scheme, the range of expression levels that a provirus can potentially experience depends on the genomic entities populating the IS^27,46,55,56^. For example, the MMEs of transcriptionally-repressed locations, such as centromeric heterochromatin, would never shift to favorable configurations, underlying a null expression potential. In contrast, MMEs at genic or intergenic regions are eventually regulated by the host to induce or inhibit local activity, underlying a variable activation potential.

## Proviral fate

Host-driven HIV expression alone is not sufficient to explain the gene-expression levels, which clearly distinguish active-replication from latency^18^. The transition between these two regimens requires the interplay of viral factors. In-vitro experiments using the LTR-GFP-IRES-Tat (LGIT) HIV model vector showed that the transition from latency to active-replication is strongly regulated by the viral protein Tat. Upon a threshold level of Tat expression, clones experience a *phenotypic bifurcation* towards either one of the two developmental fates^20^. Whereas below and above the threshold, clones stably maintain latency and active-replication, respectively^20^. Biochemical studies showed that Tat promotes HIV gene-expression bypassing the host during transcriptional and post-transcriptional steps. Tat augments its own production forming a positive feedback-loop, which amplifies viral expression up to ~100 folds^57^.

**Fig. 4:**
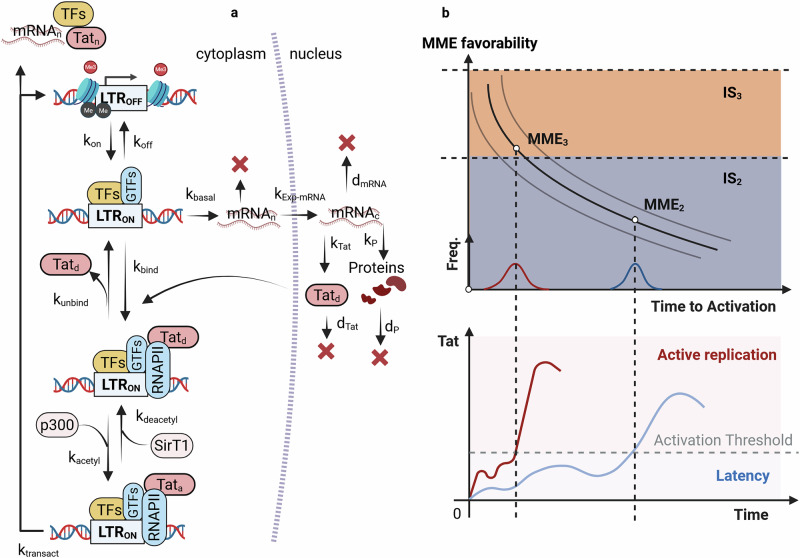
HIV gene-expression, Tat feedback-loop, and proviral activation. **a** HIV gene-expression model with Tat feedback-loop. In this model the LTR toggles between an active state *LTR*_*ON*_ and inactive state *LTR*_*OFF*_, with rates *k*_*on*_ and *k*_*off*_, respectively. An active LTR transcribes viral RNA at rate ${k}_{{basal}}$, which is exported in the cytoplasm at rate ${k}_{{Exp}-{mRNA}}$. Subsequently, viral proteins are synthesized from the mRNA at rate ${k}_{P}$, and the protein ${Tat}$ is synthesized at rate ${k}_{{Tat}}$. Simultaneously, viral transcripts, Tat and the other proteins are lost by degradation at rate ${d}_{m,}$, ${d}_{{Tat}}$, and ${d}_{P}$, respectively. Subsequently, Tat reversibly binds to the LTR at rates ${k}_{{bind}}$ and ${k}_{{unbind}}$ forming the ${Ta}{t}_{d}{LTR}$ complex. Then ${Tat}$ can be acetylated by the host histone acetyltransferase p300 at rate ${k}_{{acetyl}}$, and deacetylated by SirT1 at rate ${k}_{{deacetyl}}$. Finally, acetylated ${Tat}$ triggers transactivated HIV mRNA transcription at rate ${k}_{{transact}}$, resulting the release of Tat in its deacetylated form the LTR, where it can be eventually recycled. **b** Heuristic model illustrating the relationship between TTA and MME favorability at the proviral IS. (Top Panel) A schematic representation shows how the TTA decreases as the favorability of the MME at the proviral IS increases (black lines). Due to the intrinsic stochasticity of HIV gene expression, the TTA for each MME level is not a single value but rather a distribution—illustrated by the bell-shaped curves along the x-axis. The gray lines delineate the expected TTA boundaries for each MME, while the shaded regions indicate the range of MME favorability associated with two distinct ISs. (Bottom Panel) The lower portion of the figure displays the dynamics of Tat expression for proviruses subjected to different MME conditions, demonstrating how variations in MME favorability impact Tat-driven feedback and viral reactivation. [Created in BioRender. Palma, P. (2025) https://BioRender.com/s0hgdfm].

Stochastic models were developed to capture the Tat circuit architecture and predict proviral activation^20,25,26,34,58^. The first models [Fig. 4a] tracked the Tat-driven reactions aimed at lifting elongation blocks^20,34^, recruiting the positive transcription elongation factor (pTEFb) and nucleosome remodeling complexes (i.e., SWI/SNF)^59^. Emphasis was given to pTEFb recruitment by deacetylated Tat, and binding to the TAR element, forming and unfolding the Tat_d_ LTR complex. Then Tat is reversibly acetylated, by the host histone acetyltransferase p300^59,60^, activating, and forming Tat_a_ LTR. According to this model, Tat and pTEFb acetylation/deacetylation toggling would explain the stochastic time to activation^60^. Acetylated Tat promote transcription, then it is released in its deacetylated form and eventually recycled^61^. Coupling this Tat circuit with an *always*-*active* LTR, viral protein synthesis, and reactions of mRNA import/export, was sufficient to well predict phenotypic bifurcation in-vitro^62^.

4 $$\begin{array}{cc}\begin{array}{c}\mathrm{LTR}\mathop{\longrightarrow }\limits^{{k}_{\mathrm{basal}}}\mathrm{LTR}+{\mathrm{mRNA}}_{{\rm{n}}}\\ {\mathrm{mRNA}}_{{\rm{n}}}\mathop{\longrightarrow }\limits^{{k}_{\mathrm{Exp}-\mathrm{mRNA}}}{\mathrm{mRNA}}_{{\rm{c}}}\\ {\mathrm{mRNA}}_{{\rm{c}}}\mathop{\longrightarrow }\limits^{{k}_{{\rm{P}}}}{\mathrm{mRNA}}_{{\rm{c}}}+{\rm{P}}\\ {\mathrm{mRNA}}_{{\rm{c}}}\mathop{\longrightarrow }\limits^{{k}_{\mathrm{Tat}}}{\mathrm{mRNA}}_{{\rm{c}}}+{\mathrm{Tat}}_{{\rm{d}}}\\ \mathrm{LTR}+{\mathrm{Tat}}_{{\rm{d}}}\mathop{\longrightarrow }\limits^{{k}_{\mathrm{bind}}}{\mathrm{Tat}}_{{\rm{d}}}\mathrm{LTR}\\ {\mathrm{Tat}}_{{\rm{d}}}\mathrm{LTR}\mathop{\longrightarrow }\limits^{{k}_{\mathrm{unbind}}}\mathrm{LTR}+{\mathrm{Tat}}_{{\rm{d}}}\\ {\mathrm{Tat}}_{{\rm{d}}}\mathrm{LTR}\mathop{\longrightarrow }\limits^{{k}_{\mathrm{acetyl}}}{\mathrm{Tat}}_{{\rm{a}}}\mathrm{LTR}\\ {\mathrm{Tat}}_{{\rm{a}}}\mathrm{LTR}\mathop{\longrightarrow }\limits^{{k}_{\mathrm{deacetyl}}}{\mathrm{Tat}}_{{\rm{d}}}\mathrm{LTR}\\ {\mathrm{Tat}}_{{\rm{a}}}\mathrm{LTR}\mathop{\longrightarrow }\limits^{{k}_{\mathrm{transact}}}\mathrm{LTR}+{\mathrm{mRNA}}_{{\rm{n}}}+{\mathrm{Tat}}_{{\rm{d}}}\end{array} & \begin{array}{c}P\mathop{\longrightarrow }\limits^{{d}_{{\rm{P}}}}0\\ {\mathrm{Tat}}_{{\rm{d}}}\mathop{\longrightarrow }\limits^{{d}_{\mathrm{Tat}}}0\\ {\mathrm{mRNA}}_{{\rm{n}}}\mathop{\longrightarrow }\limits^{{d}_{{\rm{m}}}}0\\ {\mathrm{mRNA}}_{{\rm{c}}}\mathop{\longrightarrow }\limits^{{d}_{{\rm{m}}}}0\end{array}\end{array}$$

This model set the foundation to simulate and predict proviral fate in-vitro. Moreover, its analysis suggested that enhancing Tat acetylation facilitate the transitioning to active-replication^34^. However, the circuit’s intrinsic instability, which allows the two phenotypes to emerge, also causes high sensitivity to small thermal fluctuation. This sensitivity is reflected in a very low probability of observing longer times to activation (TTA), and therefore, long-term latency^25^. Real-time expression kinetics at the single-cell level showed that basal activity drives Tat pulses, suggesting the presence of some counteracting motif to Tat-amplification^25^. In the absence of any explicit repressive mechanisms, such as bi-stability and self-cooperativity, it was proposed an enzymatic resistor based on SirT1^25^. This resistor would act rapidly deacetylating Tat and competing with the p300-driven acetylation, decreasing the circuit sensitivity^60,63^. According to this model stronger resistors accompanied by weaker feedback-loop determine shorter Tat pulses following basal inputs^25,26^. Subsequent flow cytometry and single-cell imaging analyses on Jurkat cells highlighted the presence of a transient single-molecule threshold, requiring excess of inactive Tat to achieve at least one active molecule^58^. This result was modeled, introducing an additional Tat inactive state. Therefore, at its core, the Tat circuit is a non-latching positive feedback loop that generates transient pulses of expression, with the strength of the response varying with the regulatory inputs^17,18^.

According to this view, a provirus transit to active-replication—activates—when its basal activity is sufficiently high to trigger the self-maintenance of high Tat levels^20^. As discussed, the basal expression levels is stochastic and the distribution is regulated by the host through the provirus MME^27,46,55,56^. It follows that the MME at the provirus IS impacts the random TTA^55^— defined as the time required by a provirus to overcome the Tat activation threshold. A provirus exposed to a favorable MME would experience an early transition to active-replication. Conversely, proviruses with a relatively unfavorable MME would eventually activate later in time. This delay would be caused by an increased LTR toggling between active and inactive states due to increasingly significant molecular fluctuations, which lower the average transcriptional activity and increase the noise. Moreover, since the TTA is intrinsically stochastic, identical proviruses exposed to the same MME would transit to active replication at different times^20,64^.

Subsequent studies showed that Tat has other functions: it opens the chromatin at the LTR and aids GTFs recruitment, facilitating transcription initiation. Moreover, Tat promotes mRNA export and engages the cellular translation machinery, enhancing post-transcriptional steps. Direct evidence of Tat’s differential role in pediatric HIV infection during viral activation remains sparse, necessitating further investigation to uncover age-specific variation in Tat function and their implications for optimizing HIV management in children.

## HIV latency and latency reversal

The MME at the proviral IS orchestrates proviral activity. Long-term latency arises when the MME strongly represses viral expression, even though the IS potentially allows more permissive MME configurations. According to this view, latency reversal occurs when the MME is reconfigured into a permissive state. These reconfigurations can happen spontaneously, driven by host-cell reaction network dynamics, or be induced artificially through LRAs.

**Fig. 5:**
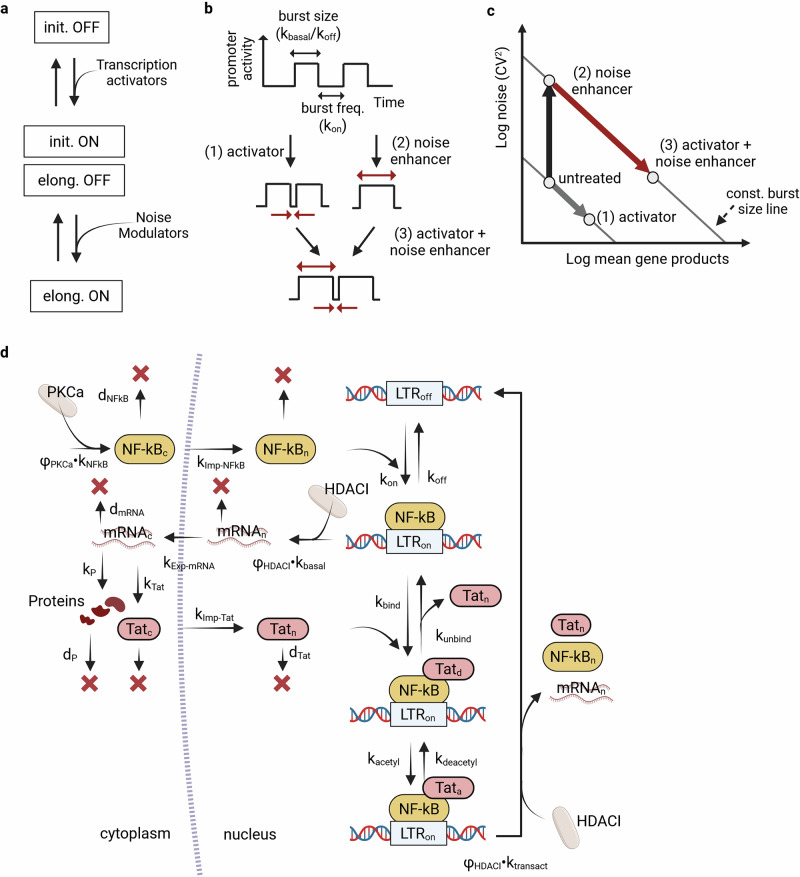
LRA synergy theory and in silico simulation of LRA-based therapy. **a** LTR state, blocks to transcriptional steps and LRAs. The schematic illustrates the key transcriptional blocks at the HIV LTR. Basal transcriptional activity is inhibited by blocks at the initiation step, while elongation blocks cause RNA polymerase pausing. In this context, LRAs that act as transcription activators are designed to overcome the initiation blocks, and the ones that act as noise modulators are intended to relieve elongation blocks. **b** HIV gene-expression modulation. HIV gene expression is characterized by bursting dynamics. Here, the burst frequency, ${k}_{{on}}$, is primarily governed by the removal or persistence of initiation blocks, whereas the burst size, ${k}_{{basal}}/{k}_{{off}}$, reflects the extent to which transcription is paused at the elongation checkpoint. **c** LRAs synergy. According to the synergy theory, when transcription activators are applied alone, they shift the system state along a diagonal representing constant burst size (path 1). In contrast, noise modulators alone increase the burst size (path 2). When combined, these LRAs act synergistically, producing an amplified increase in the average transcriptional output (path 3). **d** HIV gene-expression model explicitly considering LRAs treatment. This schematic illustrates a model of HIV transcription incorporating the modulatory effects of LRAs, specifically PKC agonists and HDACIs. In this framework, the transcription factor NFκB is released into the cytoplasm at a basal rate ${k}_{{NFkB}}$, a process enhanced by PKC agonists, ${\varphi }_{{PKCa}}$. NFκB translocate to the nucleus, ${k}_{{Imp}-{NFkB}}$ and binds the HIV LTR at rate ${k}_{{on}}$, forming the LTR-NFκB complex that initiates basal transcription, ${k}_{{basal}}$. HDACIs further amplify this transcriptional activity, ${\varphi }_{{HDACI}}$, by altering chromatin structure. The transcribed HIV mRNA is exported to the cytoplasm, ${k}_{{Exp}-{mRNA}}$ and translated into viral proteins (${k}_{P}$), including Tat, ${k}_{{Tat}}$. Deacetylated Tat is imported into the nucleus, ${k}_{{Imp}-{Tat}}$, binds the active LTR, ${k}_{{bind}}$, and undergoes acetylation, ${k}_{{acetyl}}$. The acetylated Tat-LTR complex significantly enhances transcription (${k}_{{transact}}$), establishing a positive feedback loop that drives robust viral gene expression. [Created in BioRender. Palma, P. (2025) https://BioRender.com/ygeoibo].

Initial studies on latency reversal employed *transcription activators* and *chromatin remodelers* to facilitate transcription initiation. Activators, such as TNFα and PKC agonists (i.e., prostratin and bryostatin) act as TFs enhancers and were observed to amplify mean HIV expression levels in-vitro^48^, though with limited efficacy *ex*-*vivo* and in-vivo^16^. The combined use of chromatin remodelers, such as histone deacetylase inhibitors (HDACi) (i.e., vorinostat, panobinostat, and romidepsin), methylation inhibitors, and bromodomain inhibitors has demonstrated a synergic effect, significantly enhancing treatments’ efficacy^44,65,66^. Moreover, when the intrinsic stochastic nature of gene-expression was discovered^67^, expression variability was included as a diagnostic metric^33^. This inclusion led to the discovery of *noise modulators*^33^ and the development of a theory explaining LRAs synergies^27,45^. The theory states that while transcription activators mainly act lifting transcription initiation blocks, increasing the average expression (burst frequency *k*_on_↑), noise modulators act relaxing the elongation blocks, increasing the expression noise (burst size *k*_basal_/*k*_off_↑). Therefore, each class independently modulates one transcriptional step and their combined usage lead to synergistic effect, amplifying HIV expression level [Fig. 5a–c]. This theory was validated experimentally, leading to the selection of novel LRAs^33^.

Building on detailed insights into basal and Tat-activated HIV expression dynamics, researchers have developed mathematical models that simulate the effects of latency LRAs to predict treatment efficacy^30–32,42^. These models typically extend the random telegraph framework, introducing the effect of LRAs on specific steps of the expression program. A recent model includes dose-dependent parameters that represent the nuclear levels of TFs – accounting for both their baseline release and the boost provided by PKC agonist stimulation [Fig. 5d]. In this framework, an active LTR drives transcription at a basal rate in the absence of Tat. Once Tat is acetylated, transcription is significantly enhanced. Both in basal and viral regime, HDACis stimulation further increase the transcriptional output. By coupling the stochastic promoter activity with pharmacokinetic equations, this integrative model forecast the overall reactivation probability of latent cells under different LRA dosing regimens and the kinetic patterns of viral reactivation^42^. Ultimately, such models serve as quantitative tools for optimizing “shock-and-kill” strategies in HIV cure research – enabling personalized predictions of LRA efficacy based on the specific transcriptional blocks a given provirus experiences.

5 $$\begin{array}{cc}\begin{array}{c}0\mathop{\longrightarrow }\limits^{{\varphi }_{{\rm{PKCa}}}\cdot {k}_{{\rm{NFkB}}}}{{\rm{NFkB}}}_{{\rm{c}}}\\ {{\rm{NFkB}}}_{{\rm{c}}}\mathop{\longrightarrow }\limits^{{k}_{{\rm{Imp}}-{\rm{NFkB}}}}{{\rm{NFkB}}}_{{\rm{n}}}\\ {\rm{LTR}}+{\rm{NFkB}}\mathop{\longrightarrow }\limits^{{k}_{{\rm{on}}}}{\rm{LTRNF}}\\ {\rm{LTRNF}}\mathop{\longrightarrow }\limits^{{k}_{{\rm{off}}}}{\rm{LTR}}+{\rm{NFkB}}\\ {\rm{LTRNF}}\mathop{\longrightarrow }\limits^{{\varphi }_{{\rm{HDACI}}}\cdot {k}_{{\rm{basal}}}}{\rm{LTRNF}}+{{\rm{mRNA}}}_{{\rm{n}}}\\ {{\rm{mRNA}}}_{{\rm{n}}}\mathop{\longrightarrow }\limits^{{k}_{\mathrm{Exp}-{\rm{mRNA}}}}{{\rm{mRNA}}}_{{\rm{c}}}\\ {{\rm{mRNA}}}_{{\rm{c}}}\mathop{\longrightarrow }\limits^{{k}_{{\rm{p}}}}{{\rm{mRNA}}}_{{\rm{c}}}+{\rm{P}}\\ {{\rm{mRNA}}}_{{\rm{c}}}\mathop{\longrightarrow }\limits^{{k}_{{\rm{Tat}}}}{{\rm{mRNA}}}_{{\rm{c}}}+{{\rm{Tat}}}_{{\rm{c}}}\\ {{\rm{Tat}}}_{{\rm{c}}}\mathop{\longrightarrow }\limits^{{k}_{{\rm{Imp}}-{\rm{Tat}}}}{{\rm{Tat}}}_{{\rm{n}}}\\ {\rm{LTRNF}}+{{\rm{Tat}}}_{{\rm{n}}}\mathop{\longrightarrow }\limits^{{k}_{{\rm{bind}}}}{{\rm{Tat}}}_{{\rm{d}}}{\rm{LTR}}\\ {{\rm{Tat}}}_{{\rm{d}}}{\rm{LTR}}\mathop{\longrightarrow }\limits^{{k}_{{\rm{unbind}}}}{\rm{LTRNF}}+{{\rm{Tat}}}_{{\rm{n}}}\\ {{\rm{Tat}}}_{{\rm{d}}}{\rm{LTR}}\mathop{\longrightarrow }\limits^{{k}_{{\rm{acetyl}}}}{{\rm{Tat}}}_{{\rm{a}}}{\rm{LTR}}\\ {{\rm{Tat}}}_{{\rm{a}}}{\rm{LTR}}\mathop{\longrightarrow }\limits^{{k}_{{\rm{deacetyl}}}}{{\rm{Tat}}}_{{\rm{d}}}{\rm{LTR}}\\ {{\rm{Tat}}}_{{\rm{a}}}{\rm{LTR}}\mathop{\longrightarrow }\limits^{{\varphi }_{{\rm{HDACI}}}\cdot {k}_{{\rm{transact}}}}{\rm{LTR}}+{{\rm{mRNA}}}_{{\rm{n}}}+{{\rm{NFkB}}}_{{\rm{n}}}+{{\rm{Tat}}}_{{\rm{n}}}\end{array} & \begin{array}{c}{{\rm{NFkB}}}_{{\rm{c}}}\mathop{\longrightarrow }\limits^{{d}_{{\rm{NFkB}}}}0\\ {{\rm{NFkB}}}_{{\rm{n}}}\mathop{\longrightarrow }\limits^{{d}_{{\rm{NFkB}}}}0\\ {{\rm{mRNA}}}_{{\rm{c}}}\mathop{\longrightarrow }\limits^{{d}_{{\rm{mRNA}}}}0\\ {{\rm{mRNA}}}_{{\rm{n}}}\mathop{\longrightarrow }\limits^{{d}_{{\rm{mRNA}}}}0\\ {{\rm{Tat}}}_{{\rm{c}}}\mathop{\longrightarrow }\limits^{{d}_{{\rm{Tat}}}}0\\ {{\rm{Tat}}}_{{\rm{n}}}\mathop{\longrightarrow }\limits^{d{\rm{Tat}}}0\end{array}\end{array}$$

Personalized medicine is inherently challenged by both inter-individual and intra-individual variability. This important level of heterogeneity complicates the direct applicability of traditional models to predict individual responses. To address this challenge, advanced modeling strategies must be employed^68^. One promising approach is stochastic modeling, which captures population-level variability and can simulate a wide range of patient responses. In this context, patient-specific parameterization—where models are fine-tuned for individual patients or groups with similar characteristics—proves particularly effective^69^. Training such models on rich datasets (e.g., virological, and immunological information, including high-throughput single-cell transcriptomics and proteomics) enables them to reflect the unique molecular profile of each patient. Furthermore, the use of Bayesian methodologies allows for real-time updates^70^. As new patient data become available, predictions can be refined continuously, thereby supporting the ongoing optimization of therapeutic strategies.

## Discussion and conclusions

Despite extensive research, significant gaps remain in understanding the mechanisms that regulate HIV gene-expression, particularly concerning the transition from latency to active-replication, known as viral reactivation. These gaps present substantial obstacles to the development of effective LRAs and the accurate prediction of viral rebound—defined as the resurgence of detectable virus levels—following ART interruption. One critical challenge is elucidating how extracellular stimuli impact the MMEs along host-cell DNA and modulate HIV gene-expression. Host factors, such as TFs, transcriptional machinery components (i.e., GTFs, RNAPII), and epigenetic editors, play pivotal roles in regulating the activity of the LTR, shaping its molecular configuration. However, comprehensive models that integrate how variations in MME at the provirus IS influence HIV gene-expression dynamics, such as transcriptional burst size and frequency, are still lacking. Mathematical and computational modeling plays a crucial role in bridging these knowledge gaps, providing tools to predict and simulate the behavior of latent reservoirs under different conditions. Modeling helps clarify the interactions between host factors and viral elements that govern HIV latency and reactivation. By capturing how MME variations affect transcriptional noise and activation thresholds, models allow researchers to identify key regulatory mechanisms and predict how latent proviruses transition to active-replication. This predictive capability is essential for developing personalized LRAs tailored to target high-risk proviruses based on their IS and reactivation potential.

The location of IS within the host genome, along with the nature and frequency of extracellular stimuli, profoundly affects whether a provirus goes dormant or becomes actively-replicating. Developing a comprehensive reactivation likelihood map, informed by robust modeling, that links specific MMEs to the probability of latent proviruses to synthesize Tat and transition to active-replication in response to stimuli, such as immune activation, would be invaluable. Such a map, generated through modeling, would enable clinicians to identify high-risk proviruses more prone to reactivation and tailor LRA therapies more effectively.

The MME at the provirus IS is quantitatively determined using a combination of high-throughput sequencing and advanced computational analyses. IS sequencing precisely maps the locations where HIV integrates into the host genome across different cells and tissues, providing a spatial framework. This mapping is then complemented by epigenomic profiling techniques such as ChIP-seq, which detects histone modifications indicative of active or repressive chromatin states, and ATAC-seq, which measures chromatin accessibility. Additionally, RNA-seq data help correlate these epigenetic signatures with transcriptional activity, offering insights into the activity surround the IS. Computational models integrate these multidimensional datasets to generate quantitative scores representing the MME. These scores encompass parameters like DNA methylation levels, nucleosome positioning, histone mark distributions, and the presence of regulatory elements such as enhancers or promoters. While many models have been initially validated using data from resting CD4 + T cells, recent advances allow them to be adapted to other HIV-harboring cell types, including macrophages and tissue-resident cells, by incorporating cell-specific^71^. Thus, with comprehensive data integration, these models are broadly applicable and not confined to specific cell types, enabling tailored analysis of the MME in diverse biological compartments.

Predicting viral rebound following ART interruption remains a significant challenge in HIV cure research. While viral reactivation marks the initial process by which latent proviruses become transcriptionally active, viral rebound represents the clinical manifestation of renewed virus replication and detectable levels in plasma. Current predictive models, largely based on population-level dynamics, often fail to capture the heterogeneity of proviruses within an individual’s latent reservoir. Proviruses integrated at different MME may exhibit distinct latency-reversal rates, complicating accurate predictions of viral rebound. Mathematical modeling can incorporate data on IS-specific reactivation potential into viral dynamics models, significantly enhancing predictive accuracy. These improved models can guide treatment decisions and better manage patient expectations

A deeper understanding of the mechanisms by which host factors regulate HIV transcription will enable the development of predictive models that map reactivation potential across different genomic regions. Such models will facilitate more accurate predictions of time to viral rebound and support the creation of personalized LRAs targeting high-risk proviruses. Enhanced modeling approaches also enable the development of in-vitro systems that accurately simulate latency and reactivation dynamics, providing a platform to evaluate the efficacy of novel LRAs and optimize therapeutic strategies.

In pediatric populations, the dynamics of viral reactivation differ significantly from adults due to factors such as immune system maturation. Children, especially infants, have developing immune systems characterized by higher thymic activity and a greater proportion of naïve T cells compared to adults. This influences both the establishment and reactivation of the HIV reservoir. Research indicates that early initiation of ART in children can reduce reservoir size and lower reactivation risk. However, adherence to ART remains a critical challenge; adolescents often face adherence difficulties, leading to higher rates of virological failure and increased risk of viral rebound compared to adults. Mathematical models that incorporate age-specific immune dynamics and adherence patterns are necessary to predict reactivation risk more accurately in children and to design effective interventions. The EPIICAL consortium’s study on perinatally infected infants who initiated ART early highlighted that faster CD4 T-cell reconstitution correlates with viral load resurgences, emphasizing the complex interplay between immune recovery and viral reactivation in pediatric populations^72–74^. Modeling these interactions offers critical insights into how immune recovery influences reactivation risk, guiding personalized treatment strategies and improving outcomes.

In conclusion, a thorough understanding of how host factors regulate HIV transcription, aided by advanced modeling, is crucial for creating predictive reactivation maps across various genomic regions. These models improve predictions of time-to-reactivation and guide the design of personalized LRAs to target high-risk proviruses. By integrating mathematical modeling with experimental data, researchers can develop accurate in vitro models of latency dynamics and LRA effectiveness, moving closer to the goal of HIV eradication.

## Data Availability

No datasets were generated or analysed during the current study.
